# Rare *Naegleria fowleri* meningoencephalitis diagnosed via combined molecular biology and metagenomic sequencing techniques: a case report

**DOI:** 10.1186/s40249-025-01347-z

**Published:** 2025-07-17

**Authors:** Yuanjing Kou, Jiayao Zhang, Dan Wang, Lidan Cui, Qi Sun, Yanqi Lv, Ying Liu, Zhiquan He, Yuling Zhao, Hongwei Zhang, Jun Su, Yaobao Liu, Yan Deng

**Affiliations:** 1 Henan Provincial Center for Disease Control and Prevention, Zhengzhou, Henan People’s Republic of China; 2 National Health Commission Key Laboratory of Parasitic Disease Control and Prevention, Jiangsu Provincial Key Laboratory On Parasite and Vector Control Technology, Jiangsu Provincial Medical Key Laboratory, Jiangsu Institute of Parasitic Diseases, Wuxi, Jiangsu 214064 People’s Republic of China; 3 Pediatric Intensive Care Unit (PICU), Henan Children’s Hospital Zhengzhou Children’s Hospital, Zhengzhou, Henan People’s Republic of China; 4 School of Public Health, Nanjing Medical University, Nanjing, Jiangsu 211166 People’s Republic of China

**Keywords:** Primary amoebic meningoencephalitis, *Naegleria fowleri*, Genetic analysis, Metagenomic sequencing

## Abstract

**Background:**

*Naegleria fowleri*, a pathogenic free-living amoeba, causes primary amoebic meningoencephalitis (PAM), a rare but devastating disease with acute onset, rapid progression, and > 95% mortality. Despite its rarity, the catastrophic outcomes associated with this infection underscore the critical importance of prevention. In this report, we present a rare pediatric fatality caused by PAM in China, highlighting the challenges of diagnosis and treatment.

**Case presentation:**

A 6-year-old child from Lushan County, Henan Province, developed persistent high fever, headache, vomiting, and altered mental status on December 5, 2024. After receiving ineffective local treatment, the child was transferred to the Eastern District of Henan Children’s Hospital on December 7 for further evaluation and management. Upon admission, cerebrospinal fluid was collected for laboratory analysis, and antimicrobial therapy, including amphotericin B, fluconazole, and rifampicin, was promptly initiated. Despite these interventions, the patient’s condition deteriorated rapidly, and the child succumbed to the infection on December 9.

**Conclusions:**

Clinical and laboratory findings strongly suggest that the child was infected with *N. fowleri*, resulting in PAM. Epidemiological investigation suggests possible exposure at a public bathhouse. Given the survival characteristics of the *N. fowleri* and potential habitat expansion due to global warming, this sporadic case underscores PAM's lethal potential. With mortality exceeding 95%, early recognition and prompt intervention are crucial. Clinicians should maintain high suspicion for PAM in patients with compatible symptoms, especially in regions with warm freshwater exposure.

**Supplementary Information:**

The online version contains supplementary material available at 10.1186/s40249-025-01347-z.

## Background

Pathogenic amoebae represent a diverse group of organisms widely distributed in natural and artificial environments, including soil, lakes, rivers, hot springs, inadequately chlorinated swimming pools, and water distribution systems [1]. Among these, *Naegleria fowleri*, commonly termed the "brain-eating amoeba", is a particularly virulent species [2]. This free-living amoeba invades the central nervous system (CNS) by entering through the nasal mucosa, migrating along the olfactory nerve through the cribriform plate into the cranial cavity, and ultimately causing primary amoebic meningoencephalitis (PAM) [3]. PAM is a fulminant disease characterized by acute onset, rapid progression, severe clinical manifestations, and an extremely high mortality rate (> 95%), predominantly affecting children and young adults [4]. Despite its rarity, the aggressive nature of this infection necessitates urgent clinical attention.

Currently, no rapid and reliable diagnostic methods are universally available for amoebic infections, and early symptoms of PAM—including fever, headache, vomiting, and neck stiffness, overlap significantly with those of other CNS infections [5, 6]. This clinical mimicry poses substantial diagnostic challenges, often leading to delayed recognition and suboptimal treatment outcomes. In this case report, we present a detailed analysis of the diagnostic utility of multiple detection techniques applied to cerebrospinal fluid (CSF) samples from a suspected pediatric *N. fowleri* infection. Our findings aim to inform clinicians on improving diagnostic accuracy and management strategies for this life-threatening condition.

## Case presentation

### Case summary and history

The patient presented with a history of bathing at a local bathhouse 5 days prior to symptom onset, with no reported exposure to rivers, lakes, or environments containing silt or decaying vegetation, as per the mother’s account. On December 5, 2024, the child developed sudden-onset high fever, severe headache, vomiting, and altered mental status. The laboratory findings indicate significant abnormalities in CSF parameters: lactate levels were markedly elevated at 13.510 mmol/L (normal range: 1–2.780 mmol/L), S100 protein showed levels exceeding the reference range at > 39.000 μg/L (normal range: 0–0.105 μg/L), and neuron-specific enolase (NSE) was detected at > 300.000 μg/L (normal range: 0–16.300 μg/L). These findings, combined with microbiological analysis confirming *Acanthamoeba* species infection in the CSF, suggest a severe inflammatory or infectious process affecting the central nervous system. Other clinical findings are included in Supplementary Materials. Initial evaluation and treatment at a local hospital were unsuccessful, prompting transfer to the Eastern District of Henan Children’s Hospital on December 7 for specialized inpatient care.

### Clinical course and outcome

Despite escalation of therapy with amphotericin B, fluconazole, and rifampicin, the child’s condition deteriorated rapidly. Progressive neurological decline culminated in deep coma by December 9. The family opted to withdraw treatment, and the patient was discharged against medical advice (AMA). The patient succumbed to fulminant PAM-associated multiorgan failure on the afternoon of that day.

### Pathogen genomic identification

On December 10, the Henan Provincial Center for Disease Control and Prevention collected clinical samples from the patient for further analysis. Total nucleic acid was extracted from CSF using the QIAGEN QIAamp DNA Mini Kit (QIAGEN, Hilden, Germany) with an input volume of 200 μl, followed by quality control checks. Nucleic acid extraction and initial quality assessment were performed by the Jiangsu Institute of Parasitic Diseases, while library preparation and sequencing were conducted by Novogene Co., Ltd. (Beijing, China). The workflow involved fragmentation of genomic DNA using a Covaris ultrasonicator (Covaris Inc., Woburn, MA, USA) to generate optimal fragment sizes for Illumina sequencing, followed by end repair, A-tailing, adapter ligation, size selection via AMPure XP beads (Beckman Coulter, Beverly, USA), Polymerase Chain Reaction (PCR) amplification, and purification. The finalized libraries were sequenced on the Illumina HiSeq X-ten platform (Illumina, CA, USA), yielding 10 Gb of paired-end sequencing data. Sequencing reads were analyzed using the Parasite Genome Identification Platform (PGIP), a bioinformatics pipeline developed by the Jiangsu Institute of Parasitic Diseases (accessed via: https://pgip.jipd.com:1443/f/login), which enabled taxonomic classification and identification of the parasitic pathogen through alignment and matching against the PGIP-curated parasite genome databases.

### Metagenomic next-generation sequencing (mNGS)

The metagenomic sequencing results demonstrated high-quality data, with Q20 value of 98.76% and Q30 value of 95.97%. A total of 88,932,436 raw sequencing reads were obtained, of which 2,586,082 clean reads remained after quality control filtering. Taxonomic analysis classified 171,448 reads as parasitic in origin. Subsequent taxonomic analysis identified 50,410 sequencing reads assigned to *N. fowleri*, accounting for 29.40% relative abundance, providing conclusive evidence of active *N. fowleri* infection in the CSF sample (Table 1).

**Table 1:** Top 10 parasite species identified in cerebrospinal fluid samples through read-based metagenomic analysis

Species	Mapped reads	Relative abundance(%)
*Naegleria fowleri*	50,410	29.40%
*Spirometra erinaceieuropaei*	33,917	19.78%
*Toxoplasma gondii*	14,393	8.39%
*Wuchereria bancrofti*	9785	5.71%
*Pristionchus exspectatus*	7805	4.55%
*Schmidtea mediterranea*	5644	3.29%
*Onchocerca volvulus*	3428	2.00%
*Dirofilaria immitis*	1807	1.05%
*Parapristionchus giblindavisi*	1793	1.05%
*Pristionchus pacificus*	1698	0.99%

**Fig. 1:**
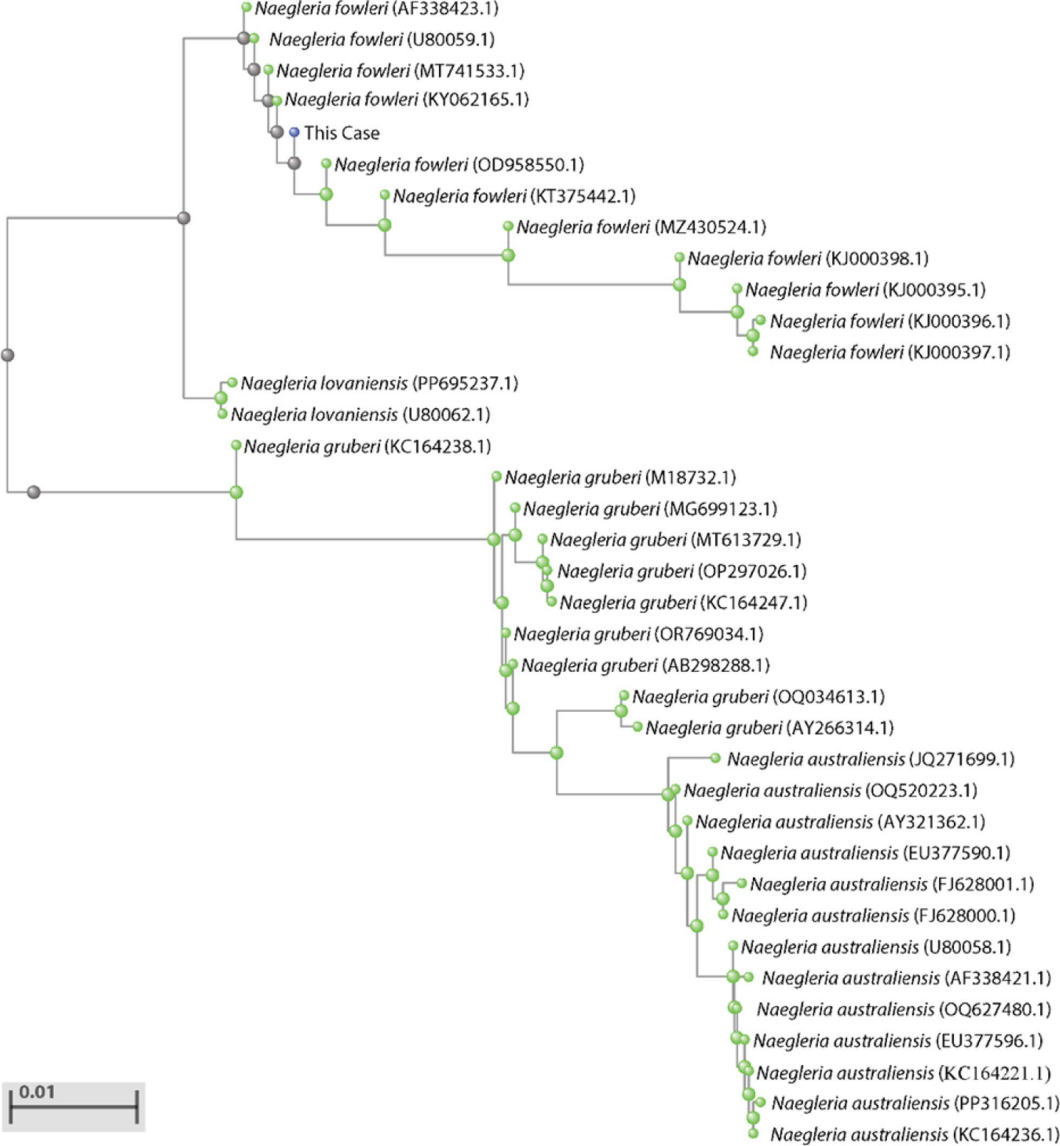
Neighbor-Joining Tree inferred from partial 18S rRNA sequences (1,843 bp) from mNGS analysis of *N. fowleri*

To further clarify the phylogenetic position of the pathogen, the sequencing data was assembled, and the 18S rRNA gene sequence was extracted for BLAST alignment analysis (NCBI, USA). Among the alignment results, the longest homologous 18S rRNA gene sequence was selected for phylogenetic tree construction. Phylogenetic analysis revealed that the pathogen in this case showed high consistency with *N. fowleri* sequences (Fig. 1).

### Targeted PCR validation

Targeted PCR was also carried out for the identification of *N. fowleri*. Gene primers were synthesized and amplified according to the protocol described in reference [7]. For *N. fowleri*, the forward primer Fwl (5'-GTGAAAACCTTTTTTCCATTTACA-3') and reverse primer RV1 (5'-AAATAAAAGATTGACCATTTGAAA-3') were used. For the *Naegleria* genus, the forward primer Fw2 (5’-GAACCTGCGTAGGGATCATTT-3’) and reverse primer RV2 (5’-TTTCTTTTCCTCCCCTTATTA-3’) were utilized. These primers were synthesized by Sangon Biotech (Shanghai) Co., Ltd.

**Fig. 2:**
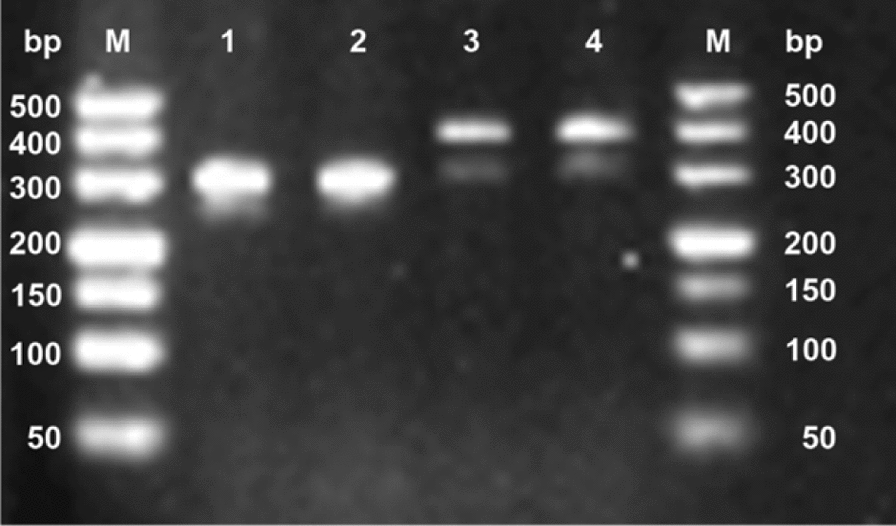
Agarose gel electrophoresis of PCR amplification products from the cerebrospinal fluid sample using species- and genus-specific primers for *Naegleria*. M, DNA marker; lanes 1–4, PCR products from the patient’s cerebrospinal fluid sample

The PCR reaction mixture consisted of 25 μl of 2 × premix, 1.5 μl each of forward and reverse primers (10 μmol/L), 5.0 μl of DNA template, and ddH_2_O to a final volume of 50 μl. The amplification products were identified by 1.5% agarose gel electrophoresis (Fig. 2) and then sent to Sangon Biotech (Shanghai) Co., Ltd. for bidirectional sequencing.

**Fig. 3:**
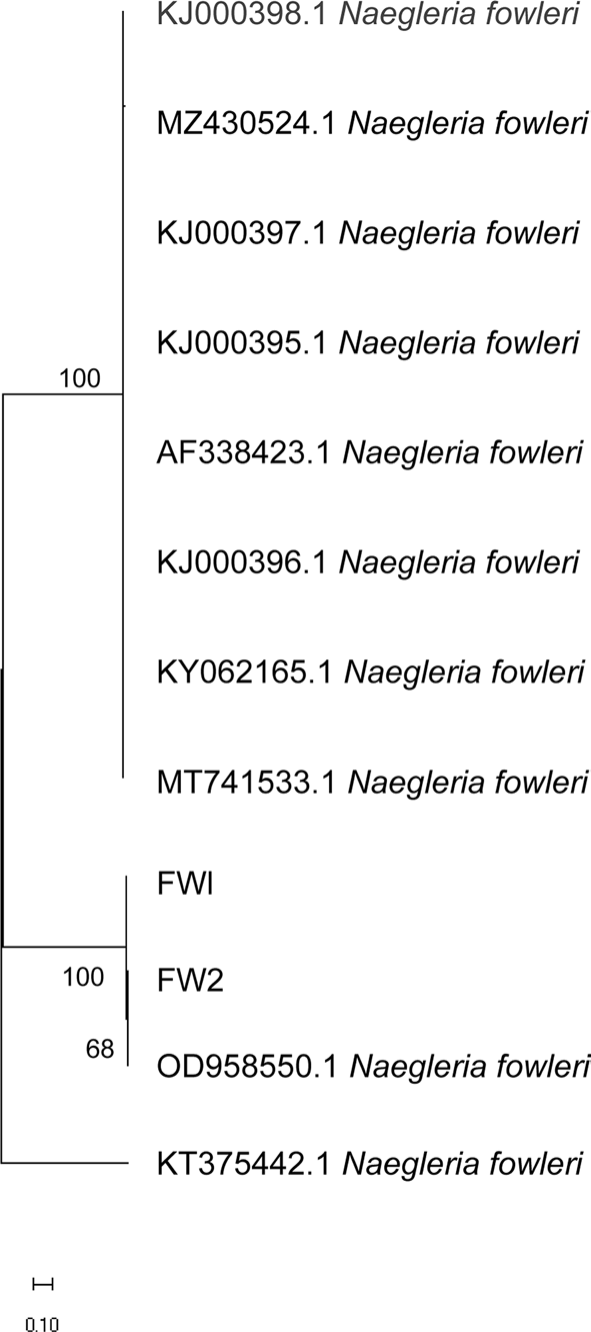
Phylogenetic tree of *Naegleria* species based on ITS gene region sequences, constructed using the NJ method. The numbers at the nodes indicate the percentage bootstrap values from 1000 replicates, with only values greater than 50% shown. GenBank accession numbers are indicated before the species names

PCR amplification results showed that the *N. fowleri gene* bands were approximately 410 bp and 310 bp in size, which matched their theoretical base pair numbers of 408 bp and 310 bp, respectively, confirming the expected fragment sizes. Sequence analysis of the amplification products via BLAST alignment confirmed both sequences as belonging to the *Naegleria* genus (Fig. 3).

### Morphological confirmation

**Fig. 4:**
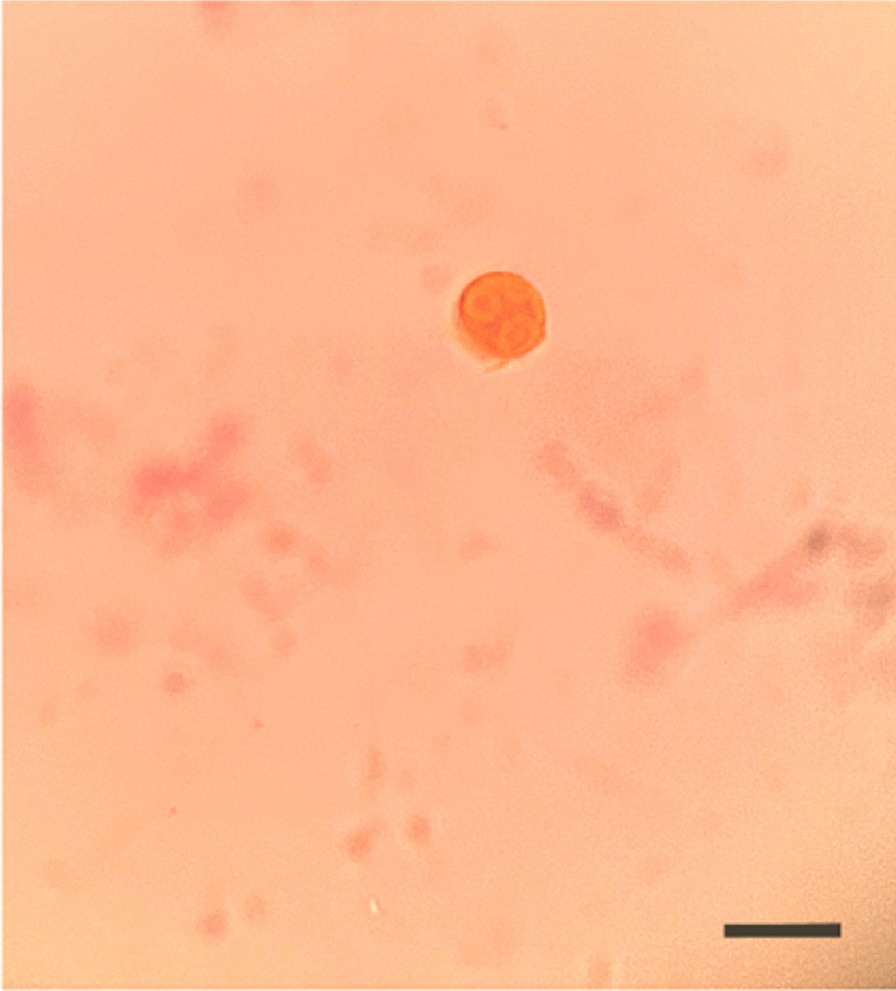
Morphology of amoebic cysts in the patient’s cerebrospinal fluid stained with iodine solution. 400 × , Scale Bar = 20 μm

Definitive morphological confirmation was achieved through microscopic examination of an iodine-stained CSF smear. At 400 × magnification, numerous *N. fowleri* cysts were identified, characterized by round morphology with diameters ranging from 15 to 18 μm and smooth, intact cyst walls. The cysts exhibited a homogeneous eosinophilic cytoplasm following iodine staining, consistent with the pathognomonic features of *N. fowleri* (Fig. 4).

## Discussion

*N. fowleri* is a thermophilic, free-living amoeba exhibiting three morphological stages: trophozoites (proliferate in 25–42 °C water), flagellates, and environmentally resistant cysts [8]. Climate warming trends are expanding the geographic and temperature ranges conducive to *N. fowleri* proliferation, elevating exposure risks during freshwater activities [9]. This study documents a fatal pediatric case of *N. fowleri*-induced PAM in China, where a 6-year-old child developed symptoms 5 days after suspected exposure at a public bathhouse and succumbed rapidly despite combined antimicrobial therapy. The fulminant disease course aligns with established pathophysiology: *N. fowleri* invades the CNS via olfactory nerves, secreting proteases and inducing contact-dependent cytolysis, which triggers irreversible cerebral edema and brainstem herniation [10–12]. Critically, the diagnosis was confirmed through a multi-technique approach: mNGS detected *N. fowleri* as the dominant pathogen (50,410 reads; 29.4% relative abundance), targeted PCR amplified species-specific ITS regions, and iodine-stained microscopy identified characteristic cysts in CSF. Despite initiating amphotericin B, fluconazole, and rifampicin after CSF analysis, treatment failed due to late intervention (> 48 h post-symptom onset), underscoring the narrow therapeutic window inherent to PAM.

Early-stage routine CSF analyses—such as elevated white blood cell counts (predominantly polymorphonuclear cells) and reduced glucose levels, lack diagnostic specificity, often leading to misdiagnosis as viral or tuberculous meningitis [13]. Further complicating diagnosis is the fact that *N. fowleri* does not grow in standard bacterial culture media, and CSF smear microscopy may fail to detect amoebic cysts due to low pathogen concentration or sampling variability. Given the rarity of cases in China, there is a lack of clinical experience in diagnosing PAM, which often results in delayed targeted treatment. In this study, we initially employed metagenomic sequencing to preliminarily identify the pathogen species, which indicated the presence of *N. fowleri* infection in the sample. Subsequently, a PCR-based detection method was used to confirm the *Naegleria* species infecting the patient. Following the methodology described by Pélandakis et al. [14], we designed two sets of primers: one specific for *N. fowleri* and another targeting the genus *Naegleria*. These primers were designed based on the ITS1-ITS2 regions. Ribosomal ITS sequences have been reported as a valuable tool for detecting inter- and intraspecific differences among various microorganisms, including *Cryptosporidium parvum*, *Naegleria* spp., and *Vahlkampfia* spp. [15–17]. Within the genus *Naegleria*, interspecies and intraspecies variations are attributed to sequence polymorphisms in the ITS2 and ITS1 regions. The ITS region has also been utilized for the identification of newly discovered *Naegleria* isolates [14, 18]. Based on the sequencing results of the PCR amplification products, it was determined that the patient had PAM caused by the rare pathogen *N. fowleri*. To further confirm the diagnosis, CSF smears were prepared using iodine staining, and microscopic examination revealed amoebic cysts, ultimately confirming the positive diagnosis.

Notably, our study demonstrates the power of integrating molecular methods for early diagnosis in resource-limited settings. mNGS identified *N. fowleri* precisely, overcoming the low sensitivity of conventional microscopy and the inability of standard cultures to support amoebic growth [19]. Subsequent ITS-targeted PCR validation provided phylogenetic confirmation of pathogen identity, while the detection of infection in a bathhouse, distinct from natural freshwater exposures, highlights emerging environmental risks requiring expanded surveillance.

Several limitations warrant mention. As a single-case report, generalizability is constrained; the absence of autopsy limited histopathological correlation, and CSF sampling during late-stage disease may have reduced pathogen detection sensitivity. Nevertheless, this approach offers a replicable diagnostic framework for rare pathogens.

## Conclusions

This fatal PAM case, one of fewer than 20 reported in China, emphasizes the lethal potential of *N. fowleri* and the imperative for rapid molecular diagnostics. The combined use of mNGS, targeted PCR, and microscopy established a diagnosis that traditional methods might miss, providing a critical roadmap for regions with limited clinical experience. Given PAM’s > 95% mortality and association with climate-warmed waters, clinicians must empirically initiate anti-amoebic therapy for patients with acute meningoencephalitis and recent warm-water exposure, even pending confirmatory tests. Concurrently, public health efforts should enforce rigorous disinfection of recreational water facilities and disseminate real-time risk alerts during high-temperature periods to prevent future tragedies.

## Supplementary Information

Additional file 1.

## Data Availability

All data generated or analyzed during this study are included in this published article.

## Abbreviations

PAM: Primary amoebic meningoencephalitis
CNS: Central nervous system
CSF: Cerebrospinal fluid
IVIG: Intravenous immunoglobulin
NSE: Neuron-specific enolase
PCR: Polymerase chain reaction
PGIP: Parasite Genome Identification Platform
