# Supplementary material for: Rare Naegleria fowleri meningoencephalitis diagnosed via combined molecular biology and metagenomic sequencing techniques: a case report
Source: Infect Dis Poverty. 2025 Jul 17;14:69. doi: 10.1186/s40249-025-01347-z (PMC12273379; doi:10.1186/s40249-025-01347-z)
Supplement: Supplementary file 1 — Additional file 1. [file 40249_2025_1347_MOESM1_ESM.docx]

**Clinical Evaluation, Interventions, and Diagnostic Findings**

Upon admission, the child received comprehensive clinical assessments of respiratory, cardiovascular, and neurological systems. Initial interventions included mechanical ventilation to manage respiratory failure, broad-spectrum antimicrobial therapy with meropenem and vancomycin to address potential bacterial co-infections, vasopressor support (norepinephrine) for hypotension, and intravenous immunoglobulin (IVIG) for immunomodulation. Multisystem stabilization efforts focused on correcting electrolyte imbalances and optimizing cerebral perfusion.

Key laboratory and imaging findings revealed significant abnormalities: urinalysis showed milky, turbid urine with a markedly positive Pandy test (+++), indicating severe proteinuria, and an elevated nucleated cell count of 14,369 cells/μL. Echocardiography demonstrated left ventricular dilation with a rounded apex, severe systolic dysfunction (ejection fraction unquantifiable), and marked hypokinesis below the papillary muscle level, consistent with myocardial injury, alongside mild tricuspid and mitral regurgitation. Abdominal ultrasound identified mild hepatomegaly, gallbladder wall thickening, and ascites, likely secondary to systemic inflammation or multiorgan dysfunction. These findings collectively highlighted a complex multisystem involvement requiring urgent and targeted therapeutic strategies.
